# Contextualizing Women’s Agency in Marital Negotiations: Muslim and Hindu Women in Karnataka, India

**DOI:** 10.1177/2158244016667450

**Published:** 2016-09-01

**Authors:** Biswamitra Sahu, Patricia Jeffery, Nakkeeran N

**Affiliations:** 1 Indian Institute of Public Health—Bangalore Campus, India; 2 University of Edinburgh, UK; 3 Ambedkar University Delhi, India

**Keywords:** agency, education, marriage, timing, negotiation, India

## Abstract

We use 36 in-depth interviews, with 18 Muslim and 18 Hindu women in Karnataka, India, to explore the relationships between women’s educational attainments and women’s exercise of agency in spousal selection and the timing of marriage. We have outlined three kinds of agency, namely, convinced, resistance, and complicit, and the contexts in which they were deployed by our participants during their marriage negotiations. Our examination of the role of education across this spectrum of agential capacities during marriage negotiations suggests that the linkages between education and agency are not straightforward. Rather, the normative context, and how parents and daughters interact with it when fixing marriages, makes the use of agency by the woman and by their parents much more complicated than standard narratives that claim that “modern” education for girls will inevitably enable women to play decisive roles in realizing their personal preferences. Our data lead us to challenge this framework and we argue that the link between education and agency is not always positive and linear, as it widely thought to be.

## Introduction

Marriages in South Asia are predominantly arranged by family elders (Caldwell, Reddy, & Caldwell, 1982; Dyson & Moore, 1983; Ghimire, Axinn, Yabiku, & Thornton, 2006; P. Jeffery & Jeffery, 1996). Kandiyoti (1988) sees South Asia as an example of “classical patriarchy” marked by the control and subordination of women, regardless of cultural and religious affiliations. Regarding marriage arrangement, Kandiyoti (1988) further adds that “girls are given away in marriage at a very young age into households headed by their husband’s father. There, they are subordinate not only to all the men but also to the more senior women, especially their mother-in-law” (p. 78).

Nevertheless, subordination can never be total, and there is always some space—albeit maybe very limited—for maneuver. In light of that, we highlight young women’s agency in the negotiations connected with their own marriages. The term *agency* has been widely used in the Global South, in large measure as a response to the portrayal of women as victims of oppressive structures. “Agency” has been seen as a way to explore the ways in which women can attain some control over their lives. There are, however, dangers in blithely celebrating women’s agency while failing to grasp the significance of how social and economic contexts influence the kinds of agency available and how women can use them. The socio-economic context affects the realistic choices that women can make, and some options apparent to outsiders are virtually “unthinkable” for the people living long-term within those structures. Furthermore, as we argue in this article, agency is not always overt and young women’s reflections and assessment of the implications of taking a particular stance may result in their appearing to go along with decisions that might seem contrary to their interests.

A woman’s preferences, for example, regarding the man chosen to be her husband and the timing of her marriage, could play an important role in determining the span and characteristics of her reproductive career.^1^ A better understanding of the context in which a woman can or cannot express her opinions about her future marriage is likely to reveal the potential she may subsequently have for influencing other aspects of her reproductive life. For instance, a woman’s say (or lack of it) during the negotiations surrounding her marriage affects her age at marriage (e.g., whether it is below or above 18, the legal age of marriage in India); the characteristics of her spouse (age, educated/uneducated, economic situation, work status etc.); and the likely quality of spousal communication (which itself eventually affects reproductive choices such as contraceptive use, family size, etc.) (Haberland, Chong, & Bracken, 2003).

While discussing female empowerment, Kabeer (1999) defines first-order choices as crucial strategies that people adopt to lead the lives that they desire, such as whether or whom to marry and how many children to have. Kabeer (1999) distinguishes between resources, agency, and achievement. Resources are a precondition that enhance a person’s ability to exercise agency and achieve a desired outcome. Resources include material, human, and social resources “which enhance the ability to make choices” (Kabeer, 1999, p. 437). Agency is the ability to set goals and achieve them.

Several scholars (e.g., Avishai, 2008; Korteweg, 2008; Mahmood, 2001) question this definition of female agency that is set a priori, because women who do not exert overt resistance against domination would be understood as lacking agency. Burke (2012) urges us to re-assess the relevance of agency in non-Western contexts. Illustrating the same point, Miller, Das, and Chakravarthy (2011) contend that agency might be constrained in collectivist cultures. Hence, understanding agency as resistance alone would fail to do justice to the range of intentional responses that actors are likely to display in non-Western contexts. Ahearn (2001) urges anthropologists to question the conceptualization of agency which may differ from society to society. She underlines the need to move away from a conceptualization of agency that is synonymous with free will or resistance. Again, the implications of the forms of resistance are often neglected (Abu-Lughod, 1990). Meyers (2000) further stresses the need to focus on the “process of deciding rather than the nature of action decided upon” (p. 470). Autonomous persons do not merely conform or resist. Rather, “they know who they are—what really matters to them, whom they deeply care about, what their capacities and limitations actually are and so forth—they enact this introspective understanding of their “true” selves in their lives” (Meyers, 2000, p. 476). Mahmood (2001) emphasizes that a woman might be required to appear *malleable*, which might suggest her passivity. Thus, Mahmood urges us to reassess “other desires, aspirations and capacities that inhere in a culturally and historically located subject” (p. 223) and further asserts that “agential capacity is entailed not only in those acts that result in (progressive) change but also those that aim towards continuity, stasis and stability” (p. 212). She introduces a different modality of agency which encompasses “ways in which women resist the dominant male order by subverting the hegemonic meanings of cultural practices and deploying them for their own interests and agendas” (Mahmood, 2001, p. 205). Korteweg (2008) advocated the inclusion of practices that do not explicitly intend to undermine hegemony into a category of “embedded” agency which still reflects “active engagement in shaping one’s life” (p. 437). Madhok, Phillips, and Wilson (2013) introduces another layer to the characterization of agency by suggesting steering the focus from maximal or free action to speech practices as a possible location for exercising agency. This broad conceptualization of agency, however, might not be useful unless the core is defined and boundaries are drawn (Brubaker & Cooper, 2000; Burke, 2012).

Several scholars, then, have shown that agency is a complex concept and they have used various labels while trying to capture its different aspects. Ahearn (2001) defines agency as “socioculturally mediated capacity to act” (p. 118). She argues for a conceptualization of agency beyond the dichotomy of either victimization or acceptance because there is always a danger that we might not account for what falls in between or “ambiguous agency” where women accept, accommodate, ignore, resist, or protest. Ahearn (2001) further urges future research to distinguish clearly typologies of complex and ambiguous agency, such as, oppositional agency, complicit agency, agency of power, agency of intention, and so on. We align with Ahearn to a large extent, and hence, using our empirical data in this article, we characterize three kinds of agency: *convinced agency* (where the actor agrees with a choice/decision, whether taken by the self or by others, is in control of the situation and is aware that he or she can take befitting action to direct the situation toward to his or her aspired outcome); *resistance agency* (Avishai, 2008; Mahmood, 2001; acts that challenge hegemonic forces and play an active role in realizing that choice); and *complicit agency*^2^ (Ahearn, 2001; acts that conform to hegemonic forces, even if the actor does not agree).

Kabeer (1999) argues that access to resources (here, education) is not automatically empowering, although it has the *potential* for transformation. Taking this discussion forward, we examine the extent to which women’s educational attainments obtained through schooling in formal educational institutions can be a potential resource for exercising agency and playing active roles in realizing choices over spouse selection and the timing of marriage. In general, more highly educated women marry later than other women. It is unclear, however, whether this delay in marriage is because education leads to agency or simply reflects the longer period such women have spent in formal education. Furthermore, empirical studies have observed that increases in female education are often accompanied by better health status (Bloom, Wypij, & DasGupta, 2001), lower fertility (Lutz, Cuaresma, & Abbasi-Shavazi, 2010), and improved nutritional and immunization status of offspring (Borooah, 2004). Jejeebhoy and Sathar (2001) have suggested that a woman’s use of agency based on her educational attainment is one route through which education influences health status. Yet the pathways through which women’s education might influence health outcomes are not well understood (Moursund & Kravdal, 2003), and the *causal* linkages through which female education might influence health outcomes are contested (Basu, 1996; P. Jeffery & Jeffery, 1996; R. Jeffery & Jeffery, 2000).

There is still a lot unknown about what if anything “education” does to empower, what we actually mean by empower, and how people make decisions (Basu, 2002, pp. 1779-1790; Batliwala, 1994; Kabeer, 1999). Crucially, moreover, women negotiate agency from their situated contexts (caste, class, religion, etc.) which channel their realistic options and thus also need to be factored in to achieve a more nuanced understanding.

## The Contours of This Article

This article seeks to develop a deeper and contextualized understanding of the processual dimensions of decision making in relation to marriage, by focusing on the contexts within which women can exercise agency. First, we outline the normative processes through which marriages are arranged. Second, we consider the potential of education as a resource for women to use agency (whether resistance, complicit, or convinced) in marital negotiations and the outcomes of such agential interventions. And we examine the differing class, caste, religious community, and location contexts in which women used agency during the negotiation of their marriages.

The data for this article were collected between 2006 and 2008, with the assistance of the Institute for Economic Research, Dharwad, and Institute for Social and Economic Change, Bangalore. Due to the exploratory nature of this research and limited time and resources, we decided to conduct in-depth interviews with 36 participants, and this discussion is based on 36 in-depth interviews, with 18 Muslim women and 18 Hindu women in Karnataka living in urban Bangalore and rural Dharwad, supplemented with some participant observation. The women represented a cross-section of caste and class backgrounds to enable us to incorporate a broad range of women’s voices. Using in-depth interviews has enabled us to build one-to-one rapport with the participants, and we present excerpts from their views that reflect shared opinions as well as unique cases.

The participants in this study were recruited according to the research objective of the lead author’s doctoral research: to explore the relevance of religion and gender for reproductive behavior. We first collected baseline data on all the women in the selected villages and urban wards, from which we purposefully sampled the study participants to include cases based on religion, age, marital, and fertility status. Our larger research aimed to understand reproductive behavior, birthing experiences, and child care practices, so we recruited currently married Muslim and Hindu women of differing ages in the prime reproductive age group (18-44 years) who already had experience of childbearing and child rearing. All the non-Muslim women were caste Hindu apart from one Scheduled Caste/*Dalit* woman.^3^ To explore the role of education in the use of agency during marital negotiations, we selected women with different educational backgrounds (see Table 1.).

**Table 1.** Distribution of Age, Education (in Years), and Residence of Recruited Participants.

	Hindu	Muslim
Age		
18-22	1	3
23-27	8	7
28-32	4	3
33-37	3	3
38-42	2	2
Years of education		
No education	3	4
1-8	4	4
9-11	6	6
12-17	5	4
Place of residence		
Rural	9	9
Urban	9	9

During fieldwork, the first author (FA) was a 26-year-old single Hindu woman and a native Oriya speaker. She had two female research assistants (RAs) then aged 23 and 24 years, who were both single Hindu women and native Kannada speakers. The interviews were based on an in-depth interview guideline. Because this article focuses on marriage negotiations it draws on only a small part of much more extensive interviews that included proximate determinants of fertility, cultural beliefs and practices surrounding pregnancy, delivery, postnatal care, nutrition, and child care, issues of religion, gender, and minority status. Apart from three interviews with Muslim participants in rural Dharwad conducted in Kannada by the RAs, FA interviewed all the Muslim participants in Hindi (which is similar to Dhakani, the language of Muslims in Karnataka). The two RAs conducted all the interviews with Hindu participants in Kannada, the language spoken by Hindus in Karnataka. Before fieldwork, FA had taken Kannada lessons and understood it fairly well, and she attended all the interviews in Kannada and could moderate the conversation when required. The interviewers are trained in using qualitative methods and they navigated the interview process with utmost care and sensitivity where direct questions are mostly avoided and interviewees were asked to narrate what had happened during marriage negotiations.

Before beginning interviews, we introduced participants to the research topic, the nature of the interview, and the use of a recording device. We assured them of the confidentiality of the information they shared. If a woman agreed to participate, she was asked for verbal rather than written consent because some participants were reluctant to give written consent as they were concerned about the possible misuse of signatures or thumb prints. In addition to the voice recording, FA or one of the RAs also took detailed notes during the interviews to ensure a back-up in case of technical snags during recording. FA transcribed and translated the Dhakani interviews while the Kannada interviews were transcribed and translated by two native Kannada speakers, one a professor of linguistics and another the registrar of our collaborating institute.

We analyzed the qualitative data by coding transcripts, labeling and categorizing concepts, connecting categories and subcategories, and integrating main categories to gain a coherent understanding of the relationships between codes, categories, and subcategories.

## Results

All the study participants regarded marriage as a source of security, especially after their parents’ demise, and they reiterated the social desirability of marrying at a socially defined correct age. Their views were entirely consonant with those expressed throughout South Asia: that a daughter remains under parental care and control until marriage and afterward is her husband’s responsibility, that marriage is essential for all woman, and that any woman who remains unmarried in her natal home beyond the marriageable age is not seen in a good light. In such a setting, parents face tremendous social pressure to get their daughters married at the socially appropriate “marriageable age” (i.e., soon after puberty) for fear of being abhorred and subjected to gossip by their extended family and the community at large for failing to fulfill their parental duties.

### The Negotiation of Marriage

Our study participants’ marriages had often been conducted according to “culturally appropriate timing” based on the participant’s demographic positioning (number of siblings, birth order, sex composition). For instance, being the eldest of four sisters resulted in early marriage for a 26-year-old Muslim participant from urban Bangalore, with 10 years of schooling. She was married at 19, which she considered too early. Here, however, her parents had to arrange for the wedding expenses and dowry for each daughter and they showed urgency in marrying their eldest daughter so that they could prepare for the subsequent daughters’ weddings. By contrast, although she was younger than her as yet unmarried brother, a 20-year-old Muslim participant with 10 years of schooling had been married at 18. She considered 25 an ideal age for marriage, but her marriage was early not to delay her brother’s marriage. For him to be married before his sister would have been socially unacceptable. Another Muslim participant narrated her experience: her father chose to marry her to an orphaned man, because doing so would earn him religious merit. Her younger sister was married early because their father wanted to perform the Hajj pilgrimage, an important duty for a devout Muslim:

My younger sister was married at the age of 16. My parents had to go on Hajj. If you have to go to Hajj, then you cannot go without fulfilling your *faraz* [duties]. You have to complete all that before going [*addā karke jāte hai*]. That is why they got her married at an early age before going to Hajj. (Muslim, 27, urban Bangalore, 10 years of schooling)

Marriage between close kin (often cross-cousins^4^) is common in Southern India (Dyson & Moore, 1983). The latest marriage figures from Karnataka reveal that marriages between close kin amount to 28% (Kuntla, Goli, Sekher, & Doshi, 2013). Close kin marriage is more common among Hindus than Muslims in Karnataka (Hussain & Bittles, 2004). Lately, there has been a substantial fall in the incidence of kin marriage in South India compared with situation reported by Dyson and Moore (Fuller & Narasimhan, 2008; Srinivasan, 2005; Srinivasan, 2015). Fuller and Narasimhan (2008) attribute this to health concerns because offspring resulting from kin marriage are more likely to suffer from congenital anomalies. Srinivasan (2005) reasons that kin marriage was one strategy to avoid the fragmentation of landholdings in Southern India but that the practice has declined recently because of the practice of dowry. She notes that dowries taken in hypergamous marriages provide greater social mobility, and this accounts for the declining popularity of cross-cousin marriages in recent times.

Among the women we interviewed, six of the 18 Hindu participants had married a close relative, either their mother’s brother or a cross-cousin. None of the Muslim participants was married to their mother’s brother, and only two were married to close kin, both to maternal uncles’ sons. Empirical data suggest that women in India married to close kin generally have less education and marry at a younger age than other women (Bittles, Grant, Sullivan, & Hussain, 2002).

One Hindu participant was married to her maternal uncle when she was 17. She said the main negotiators of the marriage were her parents. Her marriage was decided at a young age and she had grown up with that presumption, so she was perhaps less likely than other young women to think there were other possibilities or to question her parents’ decision. Her matter-of-fact comment indicates her very limited role in the marriage negotiations:

Since my birth it was decided by my parents that I have to marry my maternal uncle. (Hindu, 28, rural Dharwad, no formal education)

This example indicates how decisions taken before a girl has developed the capability to comprehend the situation can potentially limit her life-chances and her ability to shape her own life and fulfill her aspirations, whether in education or any other field. Furthermore, when normative requirements oblige parents to arrange marriages among relatives, it is difficult for a young woman, without alternatives to family life, to intervene to delay her marriage or select her spouse. For instance, a Hindu participant narrated how her young age at marriage (16 years) and her lack of *buddhī* [intellect] due to her young age jeopardized her dream of pursuing higher education and taking up a job. Her marriage was not to her liking as the groom was twice her age (32 years).

I was married young. I was studying, so I was in a different state of mind. I didn’t know the meaning of marriage. I used to think marriage means going to the in-laws’ house and eating good food. I wanted to study and then work. I wanted to help my parents.

RA: Was your consent taken?

I said no. They [sister and sister’s husband] said there is no option of saying no. They said “we like the proposal so we have decided to get you married.” They got me married before I could develop *buddhī*. I didn’t like the groom even. Even then they got me married.

RA: Did you tell them?

Yes. Yes, I told them several times, but still they got me married. They forced me into it. (Hindu, 32, urban Bangalore, 12 years of schooling plus school teacher [TCH] training)

One participant revealed the tacit pressure put on her to consent to marrying her maternal uncle’s son when we asked her to speculate about her parents’ reaction if she had wanted to marry later:

When I was 18, they sought my consent to give me in marriage. I told them that I was still studying. When I was 20, they again asked me to agree to be given in marriage. I gave my consent.

FA: If you hadn’t given your consent, what would have happened?

They might have forced me. (Hindu, 33, urban Bangalore, 12 years of schooling)

The participants’ lived experiences outlined here illustrate how various structural and normative factors play important roles in the processes of marital negotiation and in the timing of marriage: birth order, number and sex of siblings, religious practice, and norms of kin marriage. Kin marriages in particular tend to happen at such young ages that a woman cannot negotiate the timing and partner for her own marriage. The participants were expected to abide by these norms to conform to the socially approved definition of an “obedient daughter.” Their parents were expected to marry their daughters in conformity with the socially approved ideas about “marriageable age.” Unmarried women and their parents alike face gossip or bad-mouthing from family and the wider community if they digress from the prescribed behavior. More than half (20) of the study participants considered their age of marriage was earlier than they had wished.

In the following section, however, we indicate how most participants regarded education as a resource that could have given them the trajectory that they wanted for their lives. Recent literature from the region suggests that this presumption about the role of education is quite widespread. For instance, Fuller and Narasimhan (2013) have observed a reduction in gender inequality among a middle-class Tamil Brahman subcaste. The authors attribute this transition to the higher education of women which has improved their employment opportunities and has resulted in greater say in their marriage arrangement. Still (2011) contends that in their quest for social mobility, an erstwhile egalitarian Telugu Dalit community perceives higher education for women to be problematic because by pursuing higher education these young women would enjoy greater freedom which might result in hypergamy, a threat to the honor—a marker of upward social mobility.

### Resources That Could (Perhaps) Have Been Empowering

One participant expressed her anguish at having had more children (four) due to her early marriage, and she compared herself with her friends with fewer children because they were married later. She also lamented the missed opportunities resulting from her early marriage and compared her situation (being a housewife) with that of her friends who studied and had jobs:

I consider it [getting married at 18] very early. My classmates, my friends, who were studying with me got married recently and each one of them has got one son. But I have got 4 kids. This was because I got married early. Everyone studied and did something with their lives [*kuch ban-ke*] and then they got married. But I finished my SSLC in 1995 and got married in 1997. That is why I feel that my parents got me married too soon. (Muslim, 27, urban Bangalore, 10 years of schooling)

Another participant was disappointed about missing the opportunity to study because of her early marriage, which she said was due to poverty. She believed that an educated woman can have more say than uneducated women in the selection of a spouse with the attributes she desires—for her, a precondition for a good married life. She also reasoned that economic independence provides the necessary support for the woman when a marriage does not work out:

I was not interested in getting married. I was too eager to study and to do something in life. But we had financial constraints at home. We were not allowed to study. Such people [who get the opportunity to study] get married by choice. I feel if you get married and get a good life partner, then it is good. If you don’t get a good one, then life gets spoilt. In that case, it is better for the girl to earn something and stand on her own feet, become independent and only then get married. That life is better. (Muslim, 25, urban Bangalore, no formal education)

A 27-year-old Hindu woman in Bangalore with 11 years of schooling expressed similar views when she said that marriage is essential, but resented being unable to pursue higher education. She said she might have been able to achieve her full potential if she had been given the opportunity to study, but her lack of education and her marriage at the age of 18 had prevented her from doing so. A different point was made by a Hindu participant from rural Dharwad, who considered that having education could protect a woman who is married late from rumors, for instance, of being romantically involved with a man of her choice:

Various rumours spread [due to late marriage or being involved in a love affair]. In order to uphold the prestige of the parents, we marry. If the girl is educated, she doesn’t depend upon others [so she is insulated from such rumours]. (Hindu, 33, rural Dharwad, no formal education)

Here, though, it becomes imperative to examine whether education is, in practice, the resource that these participants envisaged for enhancing women’s say in the selection of their spouses and the timing of their marriages. In the following sections, we examine the role of education across the spectrum of agential capacities that our study participants used.

### Convinced Agency: Satisfaction With Marital Arrangements

Although they are a small minority, seven participants expressed satisfaction over the age of their marriage and selection of their spouse (although not necessarily about the happiness of the marriage thereafter). In other words, they had exercised convinced agency. Significantly, satisfaction about age of marriage was expressed primarily by participants who were married when aged above 18 years (six out of seven), who had studied for 9 years or more (five out of seven), and who were Hindu (six out of seven). A Hindu participant from urban Bangalore and married at the age of 22 recounted her reasoning as follows:

I was married at the right age. It [age] was right for my health and also for having children. My consent was taken.

RA: If you had not liked the proposal then what would you have done?

I would have said that I don’t like this proposal. In my house, they were very careful that the boy should be good. (Hindu, 24, urban Bangalore, 10 years of schooling)

Another Hindu participant married at the age of 23 from urban Bangalore described how her consent was taken and she expressed her satisfaction:

It was the correct age, as I did not get married early.

RA: Was your consent taken.

Yes.

RA: If you had not wanted to get married could you have intervened?

My brother sat with me and discussed the proposal and then asked me for my consent. (Hindu, 24, urban Bangalore, 9 years of schooling)

The five relatively more highly educated participants had been married when aged above 18 years. A possible pathway through which higher education related to the use of convinced agency by these five women could simply be the *delay* in getting married which enhances an educated woman’s ability to use agency. Yet the two outlier cases of convinced agency used by less educated participants indicate that matters are more complex. One Muslim woman with 5 years of education said that she was already 22, which was a “marriageable age.” The other was a Hindu woman with 5 years of education who used convinced agency because “parents know what is best for me.” Four of these women’s parents asked for their consent in marriage and this might also have contributed to their satisfaction with the marital arrangement.

### Resistance Agency: Wanting to Marry Someone Better and Later

Seven participants (two participants with no modern education and five participants with 10 and more years of education) used resistance agency, one Muslim and two Hindu women from rural Dharwad, and one Muslim and three Hindu women from urban Bangalore. This section outlines the experiences of four of these women (two Hindus and two Muslims) who had tried to use resistance agency during their marriage negotiations, but met with rather different consequences. First, we outline the cases of one Hindu and one Muslim participant who succeeded in realizing their aspirations, followed by one Hindu and one Muslim who were unable to do so. The comparisons help to reveal the circumstances under which using resistance agency is more or less likely to result in the fulfillment of the participants’ immediate *practical* interests (see Molyneux, 1985) or needs (see Moser & Levy, 1986) to marry the person of her choice.

#### Aspiration realized through agency—Case 1

The first is a Muslim woman with 12 years of schooling, including training in computers, from urban Bangalore. She worked at the reservation counter of a domestic airline in Bangalore. She earned ‘10,000 per month and exercised control over her earnings. Before she got married, she had experienced two failed marriage proposals because she was outspoken, a not-so-valued attribute for a bride:

FA: Can you elaborate on the circumstances under which your marriage was negotiated?

This was not my first proposal. There were two more proposals which were broken. First, was my mother’s brother’s son. It was completely fixed at first. It came up to engagement. But three months before the wedding it was broken. That guy did not like me. I don’t know what happened. We differ in our daily life. They are more reserved and our attitude differs. He was not comfortable with me. That was dropped. The second one also happened like that, because they were not happy with the way in which we live. They are from a village. We are born and brought up here [Bangalore]. According to them, a woman should be reserved. She should not be frank or outspoken.

She used agency throughout the negotiation of the third marriage proposal which resulted in her marriage. She attributed her attitude to life to her unique upbringing: Her father treated her as an independent individual and asked for her consent before finalizing the proposal. She was the only participant whose father asked her whether she had chosen her life partner and who had placed the onus for choosing her husband on her:

My father, he brought the proposal. He told me that there is a proposal like that. “If you like it, then we will proceed.” He left me for two days. Then I decided. He told me, “you take your decision.” He did not say anything. He asked me first, “do you have any proposal? Are you in love with someone?” I said no. Then he gave me this proposal.

She had, then, taken an active role in deciding on her life partner and had also stood by her choice against opposition from the larger family because her prospective husband’s complexion was dark. She was convinced about her decision and justified it on the basis of her prospective husband’s upbringing:

I had known them for many years and knew how they had been brought up. Otherwise I would have rejected him. Everybody [members of larger family] opposed [the match] at the time of my wedding and asked me not to get married to him as he is quite dark. But I was adamant that I want this man.

We also asked her about the appropriateness of her age at the time of marriage. She was married at the age of 21 and considered it to be the right age to be married as she had attained maturity and was mentally prepared for marriage:

FA: Was it the right age for you to get married?

Yes, I was totally mature. The way I was brought up was totally different. I had already become completely mature by 19. My father brought me up like that. I had my own thoughts. Some people will not have the maturity even now. Some of my friends of my age have still not attained it. It is because of my upbringing that I was completely mentally prepared.

#### Aspiration realized through agency—Case 2

The second case is a Hindu woman residing in urban Bangalore with 10 years schooling. She is an Adi Karnataka, a marginalized caste which is enumerated as Scheduled Caste (SC) in Karnataka. She was employed in a garment manufacturing factory at the time of her marriage and was earning ‘2,400 per month. When we interviewed her, she was not working as she had recently delivered a baby. She was the only study participant who had chosen her spouse on her own. She met her prospective husband while she was working at the garment factory. Her selection was against the norm of caste endogamy and was socially unacceptable to her husband’s kin as she was a SC and her husband belonged to the Gowda caste, a dominant caste in Karnataka. Her future in-laws expressed their disapproval of the alliance and underlined their distaste by not attending the wedding. Her own brothers also refused to attend the wedding celebration. Her wedding was a very simple affair in a temple, and solemnized in 1 day in contrast to the 3-day elaborate weddings that are common among Hindus in this area. Opposition was initially voiced by her mother when her prospective husband brought the proposal to her mother:

My mother didn’t like the proposal. She was looking for a good alliance within our caste. Several enquiries had already come. When my husband told my mother about his desire to marry me, she scolded him badly and even tried to beat him.

The repercussion of her mother’s disapproval was being locked up at home, not allowed to leave the house, and barred from meeting the young man. Both she and her prospective husband were undeterred and persisted in their choice. Finally, the participant’s parents relented and took the proposal to the groom’s parents, who rejected it. At this juncture, the participant’s mother decided to get her married to the man whom she had chosen for herself. In the absence of support from the participant’s in-laws, the participant’s mother and mother’s sister undertook the logistical and economic responsibility for arranging the marriage. The participant outlined the marriage arrangements performed by her mother:

He came to my house in just a pair of clothes and with Rs 200 in his hands. My mother arranged for all the wedding expenditure and the wedding clothes, mobile, gold chain and all were bought for him.

Displaying her agency, she also explained that she married out of love and not for monetary reasons. She outlined her husband’s good qualities to justify her choice of her partner:

I didn’t want to marry him because of his property, but I liked his nature and fell in love with him. He has a lot of patience and has good behaviour. He respects girls. He knows that women should be respected. He does not drink or have bad habits. A woman who comes to her husband’s house leaving her parents should be looked after well. He has sympathy for girls. He knows his responsibility towards a girl. I liked these qualities and his outlook.

On being asked about the appropriateness of her age at marriage, she said that it was the right age as she was 23 and was working in the garment factory. She was getting many proposals, but she opted to marry the person of her choice.

These two participants were similarly placed as they were both relatively highly educated, were employed at the time of marriage, and residing in urban Bangalore. The contextual similarity does not stop here, though, as both participants received support from their parents that seems to have helped them use resistance agency and realize their aspirations in selecting their respective spouses. The Muslim woman’s father gave her freedom in spouse selection, and supported her despite the opposition she faced from the larger family. The SC woman is an outlier in this study because she faced tremendous opposition from her own family as well as the family of her spouse; finally, with grit and determination, she obtained her mother’s support to marry her chosen partner.

#### Aspiration not realized through agency—Case 3

The third case is a 22-year-old Muslim participant with religious education. Immediately prior to her wedding, she was studying in a residential *madrasa* in rural Dharwad. She was eager to continue studying there, but was summoned back home and married to her maternal uncle’s son. She expressed her anguish because her family had lied to her when they brought her back from the *madrasa*:

When I was 15 years of age, I was sent to a *madrasa* for 6 months. They [her family] told me lies and got me back and I could not finish my study of Quran. I was very interested to finish it. They told me, “this place is no good and we will send you somewhere else.” They lied to me by telling me this, and they fixed my marriage here and got me married. Otherwise I would not have got married.

She had, however, used her agency to express her eagerness to continue her education, but her family did not heed her and they justified her marriage on the grounds that she was of marriageable age:

When I said that [expressed her eagerness to study], they told me “you have attained marriageable age. How much more do you want to study? That is enough!” Then they got me married.

She had also showed her agency by expressing her disapproval to her parents because her prospective husband was a manual laborer. Nevertheless, she succumbed to the pressure exerted by her family members after she was brought back from the *madrasa* and learned that her marriage had already been fixed. She saw no *chutkara* [respite] from the situation and expressed her helplessness, because canceling the marriage after it was finalized would be socially undesirable:

They got me back after fixing my marriage. Once they had fixed it, I cannot get *chutkara* [respite]. I had to listen to them.

She was dissatisfied with the timing of her marriage because she was not allowed to pursue the education that interested her, but she was resigned to her situation and commented that parents always have good intentions when they take decisions for their children:

Whatever father and mother do, they do it right. They do it for our *bhalaiyi* [good]. This is why I became silent.

#### Aspiration not realized through agency—Case 4

The fourth case is a 25-year-old Hindu, with 12 years schooling, including ITI (Industrial Training Institute) professional training, residing in rural Dharwad. She was brought up in her maternal grandfather’s home and she explained the background to her marriage:

Since my birth, I have been brought up in my maternal grandfather’s house. They know how I am. There was no way they could doubt my character. They know my nature. Since my birth, they had decided to take me [as daughter-in-law].

When she was 15, her grandfather and parents arranged the wedding to her mother’s brother. She considered this to be very young to be married. She expressed her disappointment about being unable to fulfill her ambition to continue studying further:

At the time of marriage, I hadn’t completed 15 years. I felt it is not the right age to marry. I wanted to continue my education. That was my ambition. I hadn’t expected I would have to end my studies and get married.

She told her parents about her desire to continue studying. They assured her that she could continue further education after marriage, but they discouraged her from revealing her reluctance to marry at that time to other people.

FA: Did you tell them [parents] about it?

I told them that I wanted to continue my studies and I didn’t want to marry.

FA: What did they say?

They told me that even after marriage I can continue to study. Nobody will prevent you from continuing to study. They told me not to express my negative feelings to anybody.

In such a situation, she complied with her parents’ decision to marry her. Maybe her young age was a barrier to asserting her wish to delay her marriage. She talked about her powerlessness and compulsion to conform to her parents’ decision as she was inexperienced in matters of marriage:

Whatever they [parents] say, we have to accept. We cannot go against their wish. We don’t have enough knowledge about matters of marriage. We have to agree with their decision.

This juxtaposition of a Muslim and a Hindu participant helps us understand how neither realized her aspirations: they both used agency first to challenge hegemonic forces but then to conform. The Muslim participant had no formal “modern” education. She reluctantly married a person who did not follow the occupation of her choice, and she was married earlier than she desired and barred from pursuing her interest in religious education. The Hindu participant had 10 years of education at the time of marriage and was also married at an earlier age than she wished. Both, however, told their family members about their interest in education and desire to delay marriage. Yet, in neither case did education enable them to achieve their aspirations. The cases are also similar because both women were married to close kin and their respective immediate families did not support them in fulfilling their aspirations. There was overt coercion in the first case while the second woman was subjected to subtle coercion. Both belonged to rural areas where social controls in the matter of marriage and pressures to comply with social norms such as consanguinity seem to be stronger than in urban areas. In both cases, the norm of consanguinity, the rural context, and (probably most importantly) the lack of support from parents and wider family seem to be the obstacles preventing these two participants from realizing their aspirations.

The two Muslim participants who used resistance agency were situated at polar extremes of the modern educational spectrum: the one from rural Dharwad had *madrasa* education alone whereas the one from urban Bangalore had 12 years of modern education with professional training in computers. Of the two Hindu participants from rural Dharwad who used resistance agency, one was illiterate and the other had 12 years of education with professional training. Of the three Hindu participants from urban Bangalore who used resistance agency, two had obtained higher secondary education, namely, 12 years and 10 years of schooling. The third participant had completed 10 years of schooling at the time of marriage, although after marriage she continued her education for another 2 years and additionally obtained the Teacher Certificate Higher (TCH) training.

Hence, we conclude that having higher education is not a necessary precondition for trying to use resistance agency during marital negotiations. Nor does higher education guarantee successful use of resistance agency. Of the seven women who used resistance agency, only the two women cited earlier were successful in marrying the person of their choice and at their preferred time of marriage, but other educated women were unable to do so.

### Complicit Agency: Assessing What is at Stake

Twenty-two of the 36 participants did not consider they were married at an appropriate age, but they had refrained from openly intervening or expressing their point of view during the negotiations that preceded marriage. They all provided justifications for such a stance.

For instance, a 27 year old Muslim participant from urban Bangalore with 10 years schooling preferred to remain silent. She did not express her views overtly because that would not conform with what is expected of women and she feared being assessed negatively:

See, I could not say yes or no, because my marriage was fixed and I had to agree. Otherwise other people will think otherwise.

FA: What was that?

They will think, “why is this girl giving us a hard time [*tang kartī hai*]? She is speaking up like this [expressing her point of view].” If I had said something then it would have hurt others, so I kept quiet.

When we asked whether her consent was taken before her marriage was finalized, she asserted that the role of the closest family members is most important and so the question of seeking her opinion did not arise:

FA: Did he [father] consult you before marriage? Had he taken your consent for this?

No, no, no!!! Not like that. They don’t ask. They just arrange marriages without asking. We cannot express our *rāzī* [consent] or *arāzī* [dissent] on what is decided. If it is decided by the elders, then it is also our *rāzī*.

This woman’s acceptance of her elders’ choice of husband and timing of marriage does not reflect an *absence* of agency because agential capacity also includes those acts which enable the status quo to persist. Rather, we interpret her stance as one of carefully weighing the pros and cons of agreeing to a marriage fixed by her parents and then doing what she thought was the “right thing” given the context. Here, she fears that the repercussions of being assertive or expressing her point of view would probably be detrimental. She was likely to earn the socially undesirable tag of disobedient daughter. She felt obliged to conform for emotional reasons, to avoid hurting the feelings of her parents and wider family. And because seeking her consent for the marriage is socially undesirable, she was expected not to play any role in her marriage settlement. Hence, the most prudent course of action in the given context was non-confrontation. This was a decision that she took and to which she adhered. By deciding to comply in the face of coercion, she did not refrain from using agency. Rather, she used agency by taking that decision even though she could have refused to comply.

In a somewhat similar case, a 33-year-old, non-literate Hindu participant residing in rural Dharwad refrained from expressing her opinion to her parents because it would have hurt their sentiments. The comparison between this non-literate Hindu participant and the educated Muslim participant enables us to begin questioning how much difference education makes to women’s use of resistance agency.

Another example is an educated Muslim participant, who was married at 18. She asserted that she remained silent during the marriage negotiations because she was not pursuing higher studies and people would possibly gossip or spread rumors if she were just sitting at home:

If I had been studying, then no one would say anything. But I did not study [did not work hard at school] and if I were to stay at home, then people would say many things. That is why I was married. (Muslim, 26, urban Bangalore, 10 years of schooling)

Another reason for remaining silent during marriage negotiations is fear. A Muslim participant was worried about the backlash that would ensue if she had expressed her opinion about being married to her kinsman:

They didn’t ask for my consent. [Giggles]. I just let it happen.

RA: If you had not consented?

They might have shouted. *Helake dhairya illri* [I did not have the courage to speak up]. (Muslim, 23, rural Dharwad, 5 years of schooling)

A similar instance of complicit agency was recounted by a Hindu participant who refrained from intervening out of fear, even though she was asked for consent:

My mother fixed the proposal. They asked me but I was very scared. My maternal uncle would have shouted at me if I had expressed my opinion. (Hindu, 22, rural Dharwad, 4 years of schooling)

Despite wanting to continue her education, a more educated Hindu participant decided to remain silent because she reasoned that her intervention in the marriage negotiations would have made no difference:

The age [of marriage] was alright, but I wanted to study. My consent was not taken. I was just told [about the marriage being fixed]. I remained silent because even if I had spoken [intervened] their decision is unalterable.

RA: If you had expressed your dissent?

Still they would have got me married. (Hindu, 27, urban Bangalore, 11 years of schooling)

These cases indicate that women may refrain from using resistance agency and opt to use complicit agency in their marital negotiations for various reasons, including normative restrictions, lack of resources, emotional/family circumstances, fear, or coercion.

The Muslim women from rural Dharwad (eight out of nine) and urban Bangalore (seven out of nine) were about equally predisposed to use complicit agency. Our Muslim participants in rural Dharwad (five had no “modern” education) were less versed in modern education compared with those in urban Bangalore (three had 10 years of education), yet they deployed complicit agency to the same extent. Hindu participants from rural Dharwad (four out of nine) and urban Bangalore (three out of nine) also used complicit agency in equal proportion (though less so than the Muslim participants). Hindu participants from rural Dharwad had relatively less exposure to modern education (three had no “modern education” and three had less than 10 years of education) compared with Hindu women in urban Bangalore, and they were more predisposed to use complicit agency. Hindu participants from urban Bangalore, with relatively higher exposure to modern education (six out of nine had 10 or more years of education), also used complicit agency, though less so than the Hindu participants from rural Dharwad.

Literature that argues that there is a positive relationship between women’s education and women’s use of agential capacity has theorized agency as women’s ability to challenge hegemonic forces (resistance agency) to realize their individual aspirations. Based on the experiences of our study participants, however, we argue that being highly educated does not necessarily result in using resistance agency during marital negotiations. Crucially, in this study, most of the Muslim participants had used complicit agency during their marital negotiations, irrespective of location and educational attainment. Complicit agency was mostly used by the less well-educated Hindu participants, yet urban Hindu participants with and without exposure to modern education also used complicit agency. Consequently, we cannot discern a clear relationship between modern education and the use of complicit agency.

## Conclusion

This study is based on qualitative data from a relatively small number of participants. Hence, we cannot generalize our findings regarding the potential of education to enhance women’s ability to exercise agency in marriage negotiation. Nevertheless, our in-depth analysis of the qualitative data is suggestive of the linkages between education and agency. None of the participants disagreed with the necessity of marriage. Most of them, however, did disagree with the timing of their marriage and the choice of husband. This article deals with these disagreements and how the participants dealt with them.

The study participants’ marriages had often been solemnized according to the normative specifications of marriage timing based on their positioning within their sibling groups, religious practice, and norms of kin marriage that tend to be at younger ages than other marriages. The participants were expected to abide by these norms to be “obedient daughters.” The participants’ parents had to get their daughters married at a “marriageable age.” Failure to conform by either side would run the risk of gossip.

Based on our study, we have outlined three kinds of agency, namely, convinced, resistance, and complicit and the contexts in which they are deployed. Our examination of the role of education across this broad spectrum of agential capacities used by our study participants during the negotiation of their marriage suggests that the linkages between education and agency are extremely complex. *Convinced* agency was used by seven relatively highly educated participants: their lengthier period of formal education resulted in their being married in their 20s rather than their teens, at ages they considered to be appropriate. For the seven women who used *resistance* agency, the outcomes were diverse. It might seem that educated women are more predisposed to use resistance agency yet the relationship between education and use of resistance agency is not so straightforward. The women in the first two cases we outlined successfully used resistance agency and realized their aspirations by marrying the man they wanted to marry—but they were the only ones among the seven who had used resistance agency *successfully*. Both received support from their parents: One stood up against wider family opposition to marry a man with a dark complexion and the other battled against the odds to marry a caste-Hindu man, despite being from a SC caste. Both women were relatively highly educated and were employed at the time of marriage. The woman in the third case also had modern education, but the fourth had no exposure to modern education. Both voiced their intention to continue education by delaying marriage, but their parents and wider family used coercion (explicit and subtle) to formalize a marital alliance with a closely related man. Neither of them used resistance agency successfully, as was also the case for the remaining three women. In other words, the outcomes of using resistance agency are rather unpredictable. The majority of participants (22) used *complicit* agency in their marital negotiations, because of normative restrictions, lack of resources, emotional/family circumstances, fear, or coercion. Many, including those who were educated, remained silent during the marriage negotiations and did not challenge the status quo. Clearly, the outcome of using complicit agency might be non-confrontational, but it has the potential to result in a marriage which is either too early or to a person who is not desirable.

Burke (2012) urges scholars to etch clear boundaries around what we mean by compliant agency (equivalent to complicit agency) to make “agency empirically and theoretically useful rather than an all-encompassing term that offers little for productive research” (p. 130). Thus, we return to the question we raised earlier: whether, in practice, education actually is a resource that can change women’s life trajectories in relation to marital negotiations. Our data challenge the assumption that modern education for women will necessarily enable women to play decisive roles in realizing their personal preferences. But we do not wish to exclude the potential of education to help young women to realize their aspirations, not least because the participants themselves perceived it as a resource. It may be that the delay due to pursuing higher education enhances women’s ability to use agency. Certainly the role of women’s education in relation to women’s agency needs to be conceptualized more subtly than just counting “number of years of schooling.” We need to consider the *quality* of educational experiences and attainment, as well as the wider context in which it takes place. The findings of our article exploring the role of women’s education in relation to women’s agency are inconclusive. There are other important considerations, however, that might have more explanatory leverage in relation to women’s use of agency. They are beyond the scope of this article, however, and we are dealing with them elsewhere.

Here, we had set out to examine the extent to which women’s educational attainments obtained in formal educational institutions might be a potential resource for exercising agency and playing active roles in realizing choices over spouse selection and the timing of marriage. The demographic literature is rife with the presumption that education induces *only* resistance agency. Our data lead us to argue that the link between education and resistance agency is not always as positive and linear as it is widely thought to be. Other factors, such as the normative context and how parents and daughters interact with it when fixing marriages, make the use of agency by the woman and their parents much more complicated, for otherwise, all the highly educated participants would have used resistance agency, which is far from being substantiated by our study.

## Notes

1. In Indian local languages and in Indian English, an unmarried woman is generally referred to as a “girl.” Similarly, an unmarried man is referred to as a “boy.” In this text, however, we use “woman” and “man” throughout, as this accords with global English usage.

2. Burke (2012) uses the term compliant agency to connote something similar to what Ahearn (2001) refers to as complicit agency.

3. Although others differentiate between caste Hindus and Scheduled Caste (SC), we included the SC woman among Hindus because SC practices—caste endogamy (marriage within same caste), marriage negotiations, and marriage rituals—are similar to those among caste Hindus.

4. Cross cousins are children of opposite sex siblings, while parallel cousins are the children of siblings of the same sex.
